# Effects of TRP channel agonist ingestion on metabolism and autonomic nervous system in a randomized clinical trial of healthy subjects

**DOI:** 10.1038/srep20795

**Published:** 2016-02-17

**Authors:** Stéphanie Michlig, Jenny Meylan Merlini, Maurice Beaumont, Mirko Ledda, Aude Tavenard, Rajat Mukherjee, Susana Camacho, Johannes le Coutre

**Affiliations:** 1 Nestlé Research Center, Vers-chez-les-Blanc, Lausanne, Switzerland; 2 The University of Tokyo, Organization for Interdisciplinary Research Projects, 1-1-1 Yayoi, Bunkyo-ku, Tokyo 113-8657, Japan

## Abstract

Various lines of published evidence have already demonstrated the impact of TRPV1 agonists on energetic metabolism through the stimulation of the sympathetic nervous system (SNS). This study presents a trial investigating if stimulation of the two related sensory receptors TRPA1 and TRPM8 could also stimulate the SNS and impact the energetic metabolism of healthy subjects. The trial was designed to be double-blinded, randomized, cross-over, placebo-controlled with healthy subjects and the impact on the energetic metabolism and the autonomic nervous system (ANS) of cinnamaldehyde, capsaicin and a cooling flavor was measured during the 90 min after ingestion. Energy expenditure and substrate oxidation were measured by indirect calorimetry. An exploratory method to measure ANS activity was by facial thermography and power spectral analysis of heart rate variability using ECG was also used. Following cinnamaldehyde ingestion, energy expenditure was increased as compared to placebo. Furthermore, postprandial fat oxidation was maintained higher compared to placebo after cinnamaldehyde and capsaicin ingestion. Similar peripheral thermoregulation was observed after capsaicin and cinnamaldehyde ingestion. Unlike capsaicin, the dose of cinnamaldehyde was not judged to be sensorially ‘too intense’ by participants suggesting that Cinnamaldehyde would be a more tolerable solution to improve thermogenesis via spicy ingredients as compared to capsaicin.

It is generally accepted that the increasing prevalence of obesity and overweight worldwide can be attributed to an imbalance between energy intake and energy expenditure^1^. To prevent from a positive energy balance leading to body weight gain, one fundamental approach would be to enhance energy expenditure. Physical activity, basal metabolic rate (MR) and diet induced thermogenesis are the three components promoting energy expenditure^2^. It has been reported that natural ingredients such as caffeine, different varieties of tea or extracts from chili peppers such as capsiate or capsaicin are able to increase thermogenesis^3^ and that the increased energy expenditure observed results from sustained sympathetic nervous system activity originating from several different mechanisms^3^.

Capsaicin (the pungent ingredient of red chilli pepper) has a non-pungent analog molecule found in the sweet pepper capsicum variety CH-19, which is called capsiate. Several published studies summarized in a review by Ludy *et al.*^4^ have shown the impact of both capsaicin and capsiate on energy expenditure and fat oxidation^5^,^6^,^7^,^8^,^9^,^10^. Capsaicin stimulates the sympathetic nervous system (SNS) and increases the energetic metabolism in humans through sensory nerve stimulation. Since capsaicin increases catecholamine secretion *via* the activation of the central nervous system^11^, it is proposed that its thermogenic effect is due to β-adrenergic stimulation of brown adipose tissue (BAT) metabolic function. Both capsaicin and capsiate are agonists of the Transient Receptor Potential Vanilloid 1 (TRPV1), a broadly tuned cation channel^12^,^13^. Thermogenic and metabolic effects of capsaicin and capsiate are hypothesised to be due to the activation of TRPV1. Indeed, capsaicin prevents obesity of mice fed with high fat diet, and evidence is provided with knock out (KO) animals that this effect is mediated by TRPV1^14^. A recent study shows that TRPV1 KO mice are more sensitive to body weight gain and become more insulin resistant under high fat diet than wild type animals (WT)^15^. Taken together these studies seem to position TRPV1 as a dominant player in the regulation of energy balance.

TRPV1 is expressed in a subset of nociceptor sensory neurons innervating the mouth and the gut, and is involved in temperature and chemical sensing. Besides capsaicin and capsiate, it also responds to numerous natural molecules (*e.g.* vanilloids, gingerol, zingerone, eugenol or piperine) as well as to both temperatures above 42 °C and low pH^16^. Within the TRP channel family, two other channels are activated by various compounds found in plants that are used as spices: TRPA1 and TRPM8. TRPA1 responds to many irritant and pungent molecules found in plants (*e.g.* mustard oil, garlic, cinnamon, cloves or ginger) as well as temperatures below 17 °C^17^. TRPM8 is activated by compounds such as eucalyptol, menthol or linalool as well as by temperatures below 25 °C^18^.

Converging evidences are showing that TRPA1 and TRPM8 may, like TRPV1, impact energy balance and metabolism. In rodents it has been observed that agonists of TRPV1, TRPA1 and TRPM8 increase thermogenesis^19^. Indeed intragastric administration of capsaicin, menthol or cinnamaldehyde in mice, increases colonic temperature^19^. It has also been shown that, in rats, intramuscular injections of either capsaicin or isothiocyanate, a TRPA1 agonist, stimulate BAT function, which is the key effector of diet induced thermogenesis^20^. Moreover, TRPA1 agonists have been shown to induce adrenaline secretion^21^. As some obese animal models are presenting impaired thermogensis^22^ BAT transplantation can reverse obesity^23^. TRPA1, as a BAT function stimulator, would therefore appear to be a good pathway to target obesity. Recently, chronic administraiton of a TRPA1 agonist, cinnamaldehyde, has been shown to reduce body weight gain and improve insulin sensitivity of diet-induced obese mice. However, no evidence was obtained related to either long term regulation of BAT activity or adrenaline secretion^24^.

TRPM8 was shown to be expressed in BAT and its stimulation enhances thermogenesis *via* an up-regulation of the uncoupling protein 1 (UCP1) and consequently reduces body weight gain of diet induced obese animals^25^. Additionally, it was shown that adipocytes are able to directly sense cold temperature and up-regulate expression of genes involved in thermogenesis such as UCP1, in a β-adrenergic independent pathway^26^.

Based on this pre-clinical evidence we hypothesized that stimulation of TRPA1 and TRPM8 pathways could, as with the stimulation of TRPV1, increase thermogenesis promoting energy expenditure and thereby exert an antiobesity effect. The primary objective of this exploratory clinical trial was to compare the impact on energy expenditure of a relevant agonist of each of the three TRP channels of interest, respectively: capsaicin, cinnamaldeyhde and a cooling flavor versus placebo. Secondary objectives were to compare their impact on substrate oxidation and on the activation of the SNS measured by heart rate variability (HRV) analysis and facial thermography as respectively gold standard and exploratory methods.

## Results

### Ingredient selection

**Figure 1:**
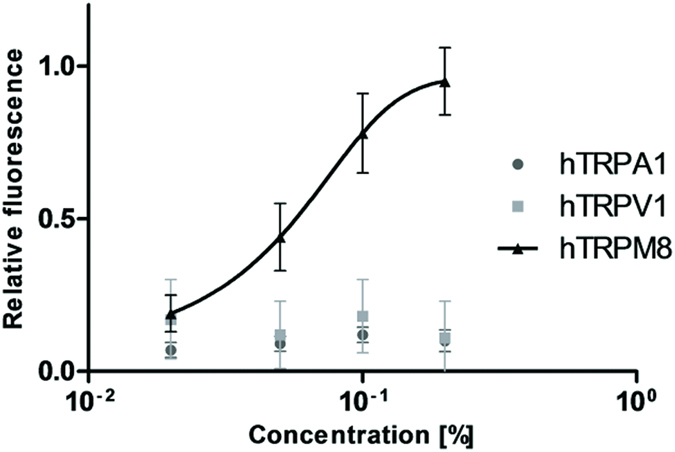
Impact of Givaudan Cooling flavor (QB-113-979-5) on hTRPA1, hTRPV1 and hTRPM8. By calcium imaging the effect and specificity of the cooling flavor has been evaluated on cells expressing hTRPA1, hTRPV1 or hTRPM8. The cooling flavor activates specifically hTRPM8 in a dose-dependent way. Data are represented by the mean of relative fluorescence ± s.e.m, n = 6. EC50 =~0.07% (=half maximal effective concentration).

The objective of this trial was to assess whether the stimulation of TRPA1 and TRPM8 pathways could be as efficient as the stimulation of TRPV1 by capsaicin or capsiate^4^ in impacting energy balance. Based on the literature, cinnamaldehyde was selected as the most representative TRPA1 agonist: since it activates specifically TRPA1 with an EC50 of 60 μM^27^. Its specificity on the human form of the receptor was confirmed *in vitro* (data not shown). To identify an efficient and specific agonist of hTRPM8, different ingredients with cooling properties were evaluated *in vitro*. Following these initial tests, a cooling flavor (CF) that specifically activates hTRPM8 with an EC50 of 0.07% (Fig. 1) was selected. As a positive control, capsaicin was evaluated in parallel to cinnamaldehyde and CF in this trial^12^.

The requirement to administrate a safe and tolerable dose determined the concentrations tested for each ingredient. Based on a safety assessment and on informal sensory evaluation these were 1 mg of capsaicin, 70 mg of cinnamaldehyde and 0.2 ml of CF in 200 ml of tomato juice. The doses represent concentrations of 16 μM, 2.6 mM and 0.1% respectively.

### Trial population

The trial utilized a cross-over design, in which all subjects who completed the study received, in different visits, all the treatments and the placebo but in a double-blinded and randomized order (see Table 1 for the randomized sequences of treatment). In total, 19 healthy subjects, all of whom were ‘moderate spicy food eaters’, were recruited and randomized; but only 16 completed the study (Table 1). Nevertheless, all of the completed sessions were included in the statistical analysis regardless of whether the subject completed the full protocol or not. In consequence, the n per treatment varies from 16 to 18 depending on the session at which the particular subject dropped-out.

**Figure 2:**
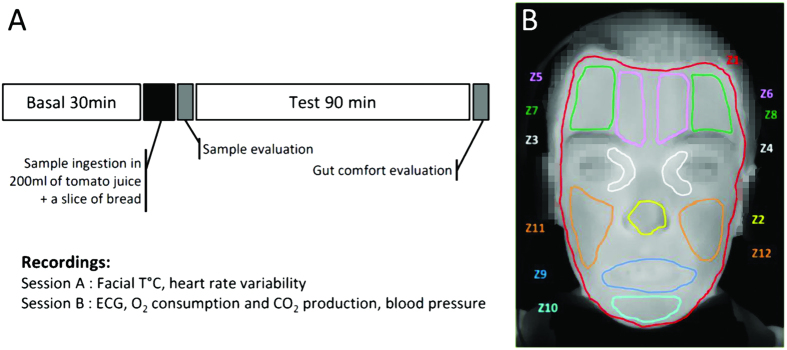
Trial design (**A**) and facial regions of interest analyzed by facial thermography (**B**).

Three subjects dropped-out: one because of discomfort, one withdrew consent and ECG signal was not measurable in one of the sessions of a third one. The population’s mean age was 32 years with a BMI of 22.43 kg/m^2^ (Table 1). Each treatment was evaluated over the 90 min following sample ingestion in two different sessions A and B, each with the same design but different recordings (Fig. 2A).

### Energy expenditure

**Figure 3:**
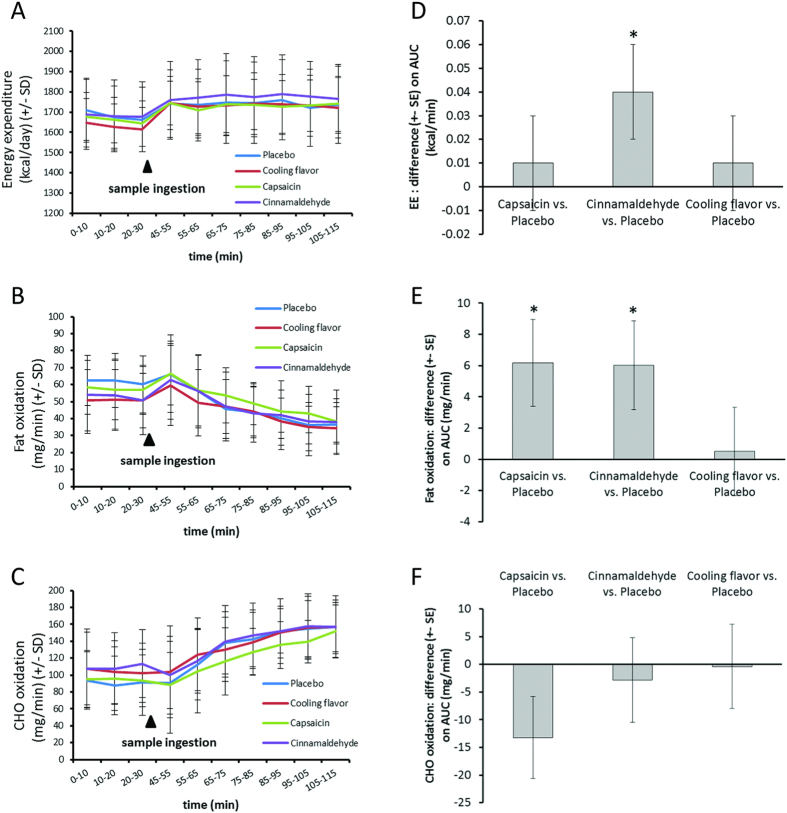
Effect of treatment on Energy expenditure (**A**,**D**), fat oxidation (**B**,**E**) and CHO oxidation (**C**,**F**). (**A**–**C**) Data distribution averaged over 10 min +- SD. (**D–F**) Comparison of treatment AUC (time standardized) +– SE. Cinnamaldehyde significantly increases AUC of energy expenditure compared to placebo (**D**). Both cinnamaldehyde and capsaicin significantly increase fat oxidation AUC compared to placebo (**E**). (*p < 0.05; Unadjusted p-values computed using mixed model, n = 16–18).

In Fig. 3, energy expenditure is represented as the distribution of the data averaged over 10 min intervals (Fig. 3A). The difference of each treatment relative to the placebo was evaluated as the time standardized area under the curve (AUC) after ingestion until the end of the session (Fig. 3D). When compared to the placebo, cinnamaldehyde increased energy expenditure (unadjusted p < 0.05), whereas no significant differences were observed for capsaicin or CF. Extrapolating over the 90 min of recording, the difference of energy expenditure between cinnamaldehyde treatment and placebo would represent 3.6 kcal (i.e. 2.4 k Cal/hr).

### Fat and carbohydrate (CHO) oxidation

Figure 3B,C shows, fat and CHO oxidation data. These are plotted as the distribution of data over 10 min intervals and the time standardized difference of each treatment compared to placebo as AUC after ingestion until the end of the session (Fig. 3E,F). A higher level of fat oxidation is observed after the ingestion of capsaicin and cinnamaldehyde compared to placebo (unadjusted p < 0.05). However, no statistically significant difference of fat oxidation was observed after the ingestion of the CF compared to the placebo. None of the treatments induced any significant changes of CHO oxidation compared to the placebo (Fig. 3F).

### Heart rate variability

For each treatment, participants went through two sessions A and B, where the administration and measurement protocol was the same but the recorded parameters were different (Fig. 2).

ECG was logged during both sessions in order to evaluate ANS activity. Three parameters were calculated using power spectral analysis of the heart rate variability collected from ECG recordings in session A and B. These were: SNS index, PNS index and the VLF/TP ratio averaged over 10 minute intervals (see supplementary Tables S1 to S6 for data distribution). Sessions A and B were analyzed both separately and pooled together. Table 2 shows the statistical analysis of the comparison of AUCs of ANS parameters, each treatment being referenced to the placebo. AUCs of PNS index, SNS index and VLF/TP were compared between groups for each session A or B and the union of both sessions.

None of these parameters showed any consistent differences over both sessions A and B, except a reduced SNS index and VLF/TP induced by the CF compared to placebo statistically significant for session B only. The respective values were: −142.944 ± 67.928 (SE), p = 0.0378 (unadjusted p-value) and −3.072 ± 1.235 (SE), p = 0.0435 (adjusted p-value) (see complete analysis on Table 2).

### Blood pressure and heart rate

**Figure 4:**
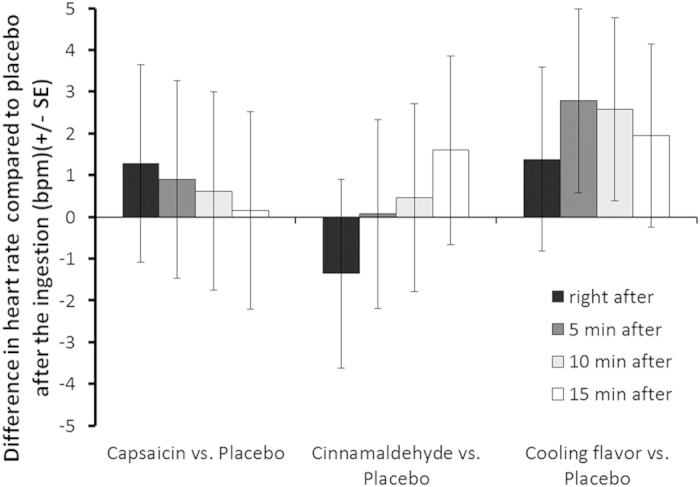
Effect of treatment on heart rate. Comparisons of different treatments with placebo at 0, 5, 10 and 15 minutes after ingestion. Data over 1 minute were aggregated and expressed as effect size +– SE. No significant differences are observed between placebo and any treatment, all p-value being >0.221 (p-values were computed using a mixed linear model, n = 16–18).

Mean arterial pressure, diastolic and systolic blood pressure (BP) were measured and calculated from the measures taken each 15 minutes during session B (time points: T0, T15, T30, T42, T57, T72, T87, T102, T117, T132) (see supplementary Tables S7–S9 for data distribution). Table 3 details the statistical analysis of the comparison of AUCs after ingestion until the end of the session of the different BP variables; each treatment being compared to the placebo. No significant difference was observed. Heart rate was measured using the ECG for each subject at each visit. To determine the effect of the different treatment, data were analyzed over 15 minutes after ingestion to capture fast changes of ANS activity. Figure 4 represents effect size of each treatment compared to placebo; no significant difference was observed.

### Facial thermography

Vasoconstriction and reduced blood flow into peripheral capillary vessels of the face, and sweating on the forehead, are phenomena that could reflect activation of the SNS under stress conditions or spicy gustatory stimulation^28^,^29^. For all products, subjects’ facial temperature was measured every second and averaged over different zones (Fig. 2B).

AUCs were compared between groups for each zone for the entire post ingestion period (Table 4). Cinnamaldehyde significantly increased chin temperature (Zone-10) compared to placebo for a prolonged time after ingestion with an adjusted p-value of 0.0486 compared to placebo.

**Figure 5:**
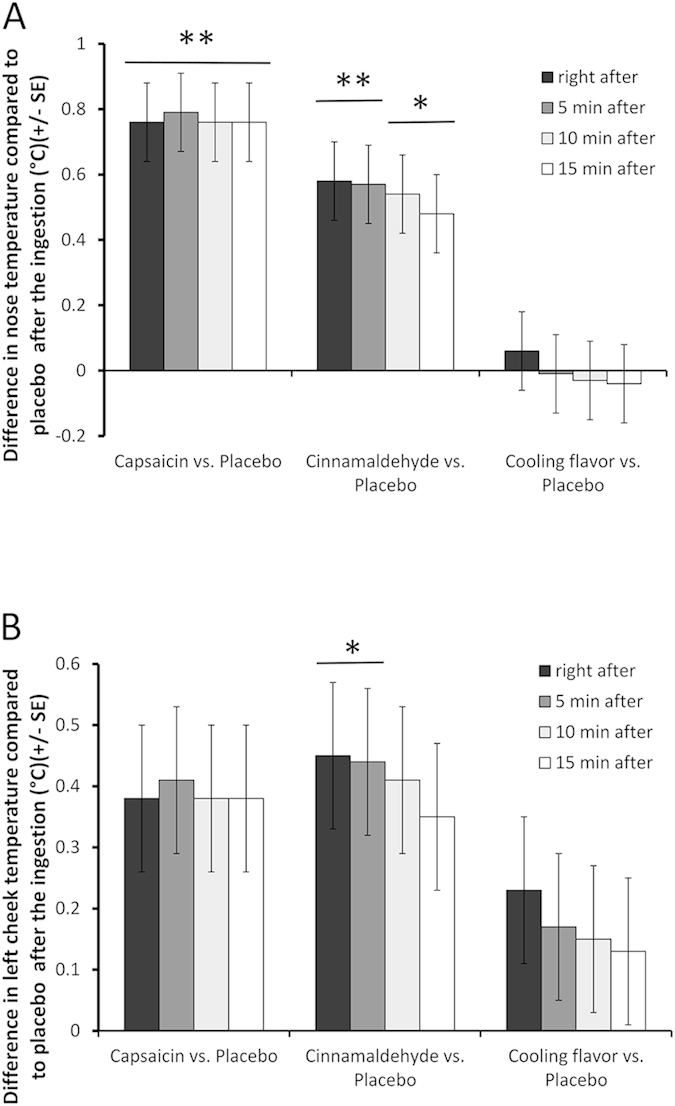
Nose (**A**) and left cheek (**B**) temperature. Comparisons of different treatments with placebo at 0, 5, 10 and 15 minutes after ingestion. Data over 1 minute were aggregated and expressed as effect size +− SE. (*Adjusted p-value < 0.05; **Adjusted p-value < 0.001; p-values were computed using a mixed linear model while adjusted for multiplicity using Holm’s method, n = 16–18).

Additionally, for each zone, data were analyzed over the 15 minute post-ingestion period in order to capture fast changes of temperature induced by product ingestion The temperature significantly increased in Zone-2 (nose) after cinnamaldehyde or capsaicin intake (Fig. 5A) and in Zone-12 (left cheek) immediately after intake of cinnamaldehyde (Fig. 5B).

### Post-ingestion evaluation of the samples

**Figure 6:**
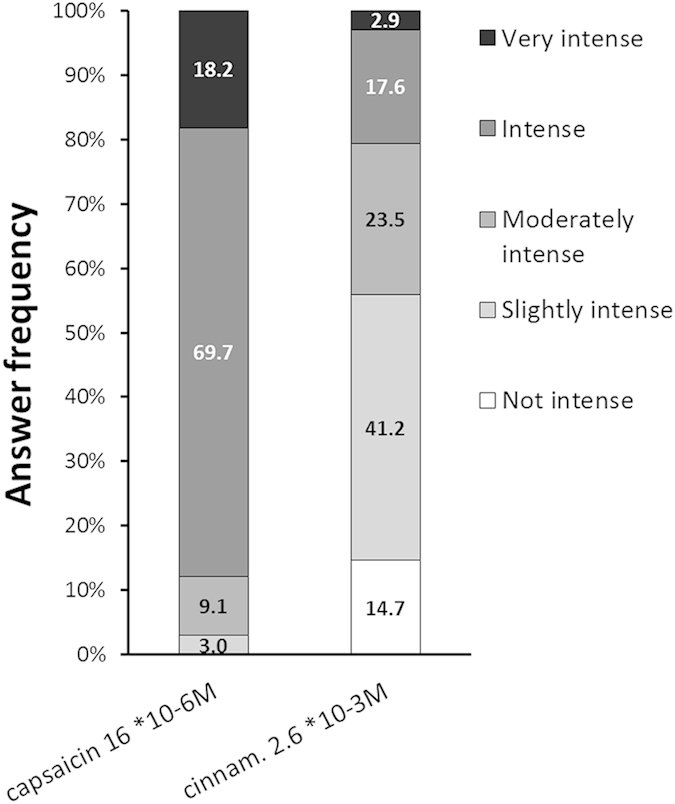
Evaluation of sample sensory intensity from questionnaire.

Intensity and chemesthetic properties of products were evaluated after ingestion using a sensory questionnaire in each session. The answers were consistent over sessions and cinnamaldehyde, capsaicin and CF could be distinguished by their sensory properties (data not shown). At the concentrations tested, cinnamaldehyde was judged less intense than capsaicin (Fig. 6) and CF was judged ‘not intense’ to ‘mildly intense’ relative to the placebo (data not shown).

## Discussion

In the present exploratory clinical trial it has been observed that a single ingestion of cinnamaldehyde (70 mg/200 ml; 350 ppm) significantly increases energy expenditure by a magnitude of about 3.6 kcal over the period of the experiment (90 min) when compared to placebo.

Moreover, both capsaicin (1 mg/200 ml; 4.88 ppm) and cinnamaldehyde induced an enhanced postprandial fat oxidation (a magnitude of about 556.2 mg and 512.7 mg respectively), over the period of the experiment (90 min) when compared to the placebo. Cinnamaldehyde increased chin temperature in the overall treatment, and both capsaicin and cinnamaldehyde increased nose temperature until 15 minute after the treatment. However, only cinnamaldehyde unilaterally increased cheek temperature in the first minutes after ingestion.

Capsaicin and capsiate, two TRPV1 agonists, have been extensively studied for their impact on weight management^4^. However, in terms of weight loss the expected magnitude of the effect produced by a hedonically acceptable dose of capsaicin is quite small^30^. Compared to the study of Ludy and Mattes^30^, in the present study, half of the dose of capsaicin, which was still perceived as intense, was used (Fig. 6). Indeed, in the study presented here, 1 mg of capsaicin had no effect on energy expenditure. Nevertheless, informal sensory testing prior to the clinical trial indicated that this dose proposed in 200 ml (4.88 ppm) was the highest acceptable. Therefore, capsaicin doses required to reach a significant effect cmetabolism would be out of the hedonic range for moderate spicy food eaters. On the other hand, the dose of cinnamaldehyde used in this study has not been judged as intense as the capsaicin one by the majority of the participants, and it gave significant results on energy expenditure compared to placebo, as well as similar results as capsaicin on fat oxidation.

The present study did not show any modification of ANS activity by heart rate variability analysis (ANS variables and heart rate). However, the increase of nose skin temperature induced by capsaicin can be interpreted as a sympathetic vasodilatation effect responding to thermogenesis to promote heat diffusion, as observed in rats by an increase of tail temperature^19^. The same effect was observed after cinnamaldehyde ingestion on nose and left cheek temperature, but the opposite effect on rat tail temperature has been reported with cinnamaldehyde and allyl isothiocyanate (AITC), another TRPA1 agonist^19^.

In the overall treatment an increase of chin temperature after cinnamaldehyde ingestion was observed compared to placebo. This temperature increase might reflect an increased blood flow in the big vessel crossing the chin. The increased blood flow could be explained by either 1) an increased cardiac output under the control of SNS, stimulated by cinnamaldehyde inducing adrenaline secretion, or, 2) by vasodilatation of this big vessel induced by the inhibition of L-type calcium channels. Indeed it was shown recently that cinnamaldehyde can inhibit L-type calcium channels independently of TRPA1, inducing vasorelaxation and decreasing blood pressure^31^.

The anti-obesity effect of long term treatment with cinnamaldehyde, as a TRPA1 agonist, in mice was not associated with any upregulation of BAT function, UCP gene expression or catecholamine secretion^24^. However, in the present study data suggest that shortly after the ingestion of cinnamaldehyde thermogenesis and SNS are stimulated. Clearly, there is a discrepancy between short term and long term treatment effects. Interestingly, the opposite effects are observed with TRPM8 agonists. In rodents it has been shown that long term TRPM8 activation with menthol increases resting metabolic rate, inducing thermogenesis via up-regulation of UCP-1 in brown adipose tissue^25^; whereas the CF, activating TRPM8 efficiently *in vitro*, does not impact energy expenditure in the present study. It may be speculated that the stimulation of the two cold sensing TRPA1 and TRPM8 pathways may lead to different and complementary responses, a rapidly induced thermogenesis and a long term adaptation of resting metabolism.

Recently, Gregersen *et al.*^32^, published a study where they measured, with comparable design, the effect on diet induced thermogenesis, appetite, energy intake and energy balance of raw spices (mustard, horseradish, black pepper and ginger) ingested as part of a meal. They observed a tendency of mustard to increase diet induced thermogenesis measured by indirect calorimetry. As mustard contains AITC, it can be suggested that the effect they observed is due, as proposed for cinnamaldehyde in the present study, to the stimulation of TRPA1 in peripheral sensory fibers and the subsequent sympathetic nerve activity, catecholamine secretion and thermogenesis stimulation. However, in the Gregersen *et al.*^32^ study, they did not observe any effect on catecholamine. Possibly the small effect they observed is due to the fact that they use raw spices instead of isolated active ingredients.

Taken together, the present results indicate that cinnamaldehyde would be a more tolerable solution to improve thermogenesis *via* spicy ingredients than capsaicin. The present concentration of cinnamaldehyde (350 ppm) is in the range of its occurrence as a flavoring in food. Cinnamaldehyde potentially is more powerful than capsaicin at improving energy expenditure and fat oxidation through thermogenic effects. Even though the effect appears subtle, a cumulative approach (combining dietary, exercise and behavioral aspects) is believed to be the most efficient for sustainable weight loss or maintenance.

Further confirmatory as well as long term studies are needed to assess the impact of a chronic ingestion of cinnamaldehyde or TRPA1 agonists on metabolism, insulin sensitivity and body weight.

## Materials and Methods

### Participants

Healthy men between 20 and 50 years old with 19 < BMI < 25 and weight greater than 60 kg were enrolled in the study. Sample size was determined to be sufficient with 15 subjects, in a cross-over exploratory study to calculate effect size. Screening of candidates was done through a questionnaire on spicy food habits to select moderate spicy food eaters. The clinical trial was approved by a competent and independent ethics committee (Commission Cantonale (VD) d’Ethique pour la Recherche sur l’Etre Humain) and participants gave their written informed consent. This trial has been conducted according to the principles and rules laid down in the Declaration of Helsinki and its subsequent amendments.

### Trial design

The study design was a double-blinded, randomized, cross-over, placebo-controlled, exploratory clinical trial. Randomization lists were produced using William’s design for a cross-over study using R version 2.8.1. The order of sessions was randomized and balanced for each subject and was determined at the beginning of the study by the statistician. The order of sessions was given for each subject to the person responsible of sample preparation who was the only person to know the order of the sessions and treatments and a code for each sample was applied. The person performing the recording was different from the person preparing the product.

For each measure, subjects were tested after an overnight fast. They were requested to use their car or public transportation to come to the investigation site in order to keep morning physical activity levels as low as possible. Moreover, consumption of caffeine has not been permitted after the lunch meal (12 a.m.). Furthermore, subjects were asked to refrain from spice consumption 2 days prior to each session. Each sample was evaluated in two different sessions A and B, with the same design but different data recording (Fig. 2A).

Each session started at 8:00 a.m. with a 30 minutes resting period and measurement preparation. During all the testing sessions subjects were seated comfortably. Room temperature was in the 20 °C to 22 °C range. Subjects were instructed to remain quiet and still, but they were allowed to watch TV. Parameters were recorded 30 minutes before and 90 minutes after ingestion of each sample (Fig. 2A). The clinical trial was registered in Clinicaltrials.gov ID: NCT02193438.

### Treatment

All active TRP agonists were dissolved in 200 ml liquid tomato juice (Granini, Eckes-Granini, Henniez, Switzerland) at room temperature and served with a slice of bread. Placebo was 200 ml commercial tomato juice. Active ingredients per 200 ml were 1 mg of capsaicin (Spectrum chemicals, Gardena, CA, USA), 0.2 ml of cooling flavor (QB-113-979-5; Givaudan, Dübendorf, Switzerland) or 70 mg of cinnamaldehyde (Sigma-Aldrich, St-Louis, MO, USA). All ingredients were *in vitro* validated in cell lines expressing hTRPV1, hTRPA1 or hTRPM8 according to the calcium imaging method described in Riera *et al.*^33^.

### Indirect calorimetry

Energy expenditure was measured by indirect calorimetry using a ventilated canopy connected to a MAX-II gas analyzer (Max II metabolic system, AEI technologies, Naperville, IL, USA). The equation used by this device to evaluate energy expenditure was the simplified Weir formula^34^.

REE = (3.941 x VO_2_) + (1.106 x VCO_2_), where:

EE = energy expenditure (kcal/min)

VO_2_ = rate of oxygen consumption (l/min) and

VCO_2_ = rate of carbon dioxide production (l/min)

After having answered questions from the compliance questionnaire participants were comfortably placed in semi-recumbent position in a reclining bed, at an angle allowing the participant to ingest the test solution without having to adjust position between metabolic rate measurement periods and ingestion. A canopy was installed over participant’s head. Tests started ~08:30 a.m. following a stabilization period allowing FeCO_2_ (expired fraction of CO_2_ in %) values to reach physiologic range.

Substrate oxidation (carbohydrates and fat utilization) and the respiratory quotient (RQ) were calculated from the VO_2_ and VCO_2_ values obtained with the MAX-II gas analyzer during the same timeline than energy expenditure. VO_2_ and VCO_2_ values were corrected for protein oxidation. The following equations were used to calculate the amount of fat and carbohydrates oxidized^35^:

PO = (0.8xbody weightx1000)/1440

NPVO_2_ = VO_2_-POx1.01031

NPVCO_2_ = VCO_2_-POx0.84361

FAT = (NVPO_2_-NVPCO_2_)/(2.01494x(1-0.707))

CHO = (NVPCO_2_-0.707x NVPO_2_)/(0.82821x(1-0.707))

RQ = VCO_2_/VO_2_

PO = protein oxidation (mg/min),

NPVO_2_ = non protein O_2_ consumption (ml/min),

NPVCO_2_ = non protein CO_2_ consumption (ml/min),

FAT = fat oxidation (mg/min) and

 CHO =carbohydrate oxidation (mg/min)

RQ = Respiratory quotient

### Power spectral analysis of heart rate variability

To evaluate the activity of the autonomic nervous system (ANS) power spectral analysis of the inter-beat interval (R-R) on electrocardiogram recordings (ECG) was used. The ECG signal was obtained from electrodes (Biocom 5000 Bluetooth ECG Recorder, Poulsbo, WA, USA) placed in the CM5 position on subjects. The monitored signal was computed using the HRVLive! Software (Biocom Technologies, Poulsbo, WA, USA). Periodically fluctuating components included in the HRV and their amplitudes were calculated over time into a power spectral curve. To evaluate the activity of ANS, the analysis was performed at a very low frequency (VLF) range (0.007–0.04 Hz), low frequency (LF) range (0.04–0.15 Hz), high frequency (HF) range (0.15–0.4 Hz) and the total power (TP) (0.007–0.4 Hz). HF reflects parasympathetic (PNS) influence on heart beat; LF displays mainly sympathetic (SNS) influence and partly vagal activity^36^. VLF reflects SNS activity related to energy metabolic regulation. VLFs are increased through thermogenic stimuli such as cold exposure and food or spicy food ingestion^5^,^37^.

Indices of SNS and PNS activities were calculated as:

SNS index = (VLF + LF)/HF

PNS index = HF/TP

VLF and VLF/TP are defined as the absolute and relative thermogenic SNS activities. Heart rate data (bpm) were also extracted from this recording and analyzed for the 15 minutes after ingestion.

The ECG signal was recorded continuously. ANS activity was analyzed during the 30 minutes before sample ingestion and the 90 minutes post-sample ingestion.

### Blood pressure

Systolic (SP) and diastolic (DP) blood pressure were measured every 15 minutes before the ingestion (T0, T15, T30) and after (T42, T57, T72, T87, T102, T117, T132) by a nurse, using a standard blood pressure monitor (inflatable arm cuff) during same session as the indirect calorimetry measure. Mean arterial pressure was calculated as: MAP = SP/3 + 2DP/3.

### Facial thermography

Infrared thermal imaging of the face was used as an exploratory, non-invasive recording method of sympathetic nervous system activity. Using a FLIR A325 infrared camera (FLIR Systems, Inc, Emitec AG, Rotkreuz, Switzerland) subjects were continuously recorded at a distance of ~1 meter. Thermographic imaging software, FLIR ResearchIR (FLIR Systems, Inc) was used to acquire images. The recording was performed by acquiring 1 image/second.

### Infrared Image analysis

Facial thermography data were extracted using in-house algorithms developed within the Matlab framework (The Mathworks Inc., Natick, MA, USA). First, a graphical user interface (GUI) was created to help defining manually a temperature threshold highlighting primarily subject’s faces. An alignment of the face was then computed and twelve regions of interest (ROIs) drawn manually using the GUI (Fig. 2B). Face alignment/orientation correction was computed image by image using a binary mask based on manually set temperature thresholds. Holes in the binary mask were filled and the region with the greatest contiguous area was selected. The selected region centroid was determined using the built-in Matlab region properties tool (regionprops). With the same tool, an ellipse was fitted within the region to obtain the angular orientation of the ellipse’s major axis compared to the x-axis. The original image was then shifted to center the region centroid at the origin (0,0) and rotated at an angle aligning the major axis of the fitted ellipse to the y-axis. Face alignments were computed for all images of the recorded session and average temperatures within ROIs extracted. Alignment performance was checked visually over the whole recorded session and discrepancies corrected by re-setting new regions and re-extracting the data in extreme cases (*e.g.* if a subject showed strong forward or background head movements not captured by the alignment algorithm).

### Data management

A paper case report form (CRF) was used. All data required in the protocol and captured by the investigator were transcribed on the CRFs. All questionnaires were joined to the CRF.

Indirect calorimetric data was automatically generated by the MAX-II program on an excel sheet. Heart rate power spectra analysis data (VLF, LF, HF, TP, HR for each second of recording) was exported into an excel file automatically by the HRV Live! program. Facial area temperatures were analyzed as described above and exported into an excel file. All generated data excel files were downloaded into the clinical data management system (CDMS, ClintrialTM).

### Statistical analysis

For **energy expenditure, fat and CHO oxidation**, the raw data was first smoothed (using loess) then the time-standardized AUC were calculated as the integral over the time after ingestion until the end of the experimental session divided by the length of this time interval. All analyses were carried out using linear mixed-effects models with a random subject-wise intercept to take into account the correlation between repeated measures.

For **ANS variables, blood pressure and thermography** the area under the curve (AUC) was computed after ingestion for each subject. AUCs were then compared between groups considering a linear mixed-effects model with the baseline measurement and the treatment group as fixed effects and the subject as random effect. For ANS parameters, the session was added as a fixed effect and an overall F-test was computed in order to compare the groups over the sessions. P-values were adjusted for multiple comparisons according to Bonferroni.

Additional analyses were performed to evaluate short term (within 15 minutes) effects of treatments on facial temperature and heart rate. To analyze fast changes, data were aggregated over 1 minute for before, right after ingestion (0–1 minute), after 5 minutes, after 10 minutes and after 15 minutes. Outliers were removed before aggregation. All analyses were carried out using linear mixed-effects models with a random subject-wise intercept to take into account the correlation between repeated measures. For facial temperature analysis, P-values were computed using a linear mixed-effects model while adjusted for multiplicity using Holm’s method.

### Sample sensory evaluation and gut comfort evaluation

Pleasantness and intensity of the chemesthetic stimuli was evaluated during the ingestion period using a questionnaire. Similarly, a questionnaire was used to evaluate gut comfort after the recording session.

### Trial registration

Clinicaltrials.gov ID: NCT02193438 “Physiologic Effect of Spices Ingestion” registered on July 2014.

## Additional Information

**How to cite this article**: Michlig, S. *et al.* Effects of TRP channel agonist ingestion on metabolism and autonomic nervous system in a randomized clinical trial of healthy subjects. *Sci. Rep.* **6**, 20795; doi: 10.1038/srep20795 (2016).

## Supplementary Material

Supplementary Information

## Figures and Tables

**Table 1:** 

Variable	Overall	Different randomized sequences of treatment exposure			
		Pl-Cap-CF-Cin	CF-Pl-Cin-Cap	Cap-Cin-Pl-CF	Cin-CF-Cap-Pl
Number of subjects randomized/sequence	19	5	5	5	4
Number of subjects completed/sequence	16	5	4	4	3
Drop-out	3	0	1	1	1
Age (years)	32(7.55)	35(9.9)	29.4(6.43)	31.2(9.12)	32.5(4.2)
Weight (kg)	73.74(8.26)	74.6(8.73)	71.6(6.39)	72.2(3.56)	77.25(14.38)
Height (cm)	181.11(8.35)	180(11.05)	180.8(4.55)	180.6(8.26)	183.5(11.27)
BMI (kg/cm^2^)	22.43(1.51)	23.02(1.72)	21.88(1.76)	22.16(1.33)	22.73(1.38)

Demographic data.Abbreviations: Pl, placebo; Cap, capsaicin; Cin, cinnamaldehyde; CF, cooling flavor. Age, weight, height are expressed as mean (SD), for the n of subject randomized.

**Table 2:** 

	Comparisons	Difference	SE	Raw p-value	Adjusted p-value
**PNS index**	Cap vs Pl Session A	−0.21	0.889	0.8137	1
	Cap vs Pl Session B	1.451	0.932	0.1224	0.3672
	Cin vs Pl Session A	0.909	0.889	0.3091	0.9273
	Cin vs Pl Session B	0.36	0.896	0.6891	1
	CF vs Pl Session A	0.3	0.892	0.7375	1
	CF vs Pl Session B	1.749	0.897	0.0539	0.1617
	Cap vs Pl Overall*				0.2931
	Cin vs Pl Overall*				0.5495
	CF vs Pl Overall*				0.1462
**SNS index**	Cap vs Pl Session A	57.399	104.905	0.5855	1
	Cap vs Pl Session B	−165.494	70.72	0.0212	0.0636
	Cin vs Pl Session A	−76.996	104.976	0.465	1
	Cin vs Pl Session B	−74.064	67.727	0.2767	0.8301
	CF vs Pl Session A	−80.111	104.85	0.4466	1
	CF vs Pl Session B	−142.944	67.928	0.0378	0.1134
	Cap vs Pl Overall*				0.0682
	Cin vs Pl Overall*				0.3638
	CF vs Pl Overall*				0.0617
**VLF/TP**	Cap vs Pl Session A	0.605	1.429	0.6728	1
	Cap vs Pl Session B	−2.135	1.284	0.0996	0.2988
	Cin vs Pl Session A	0.608	1.43	0.6715	1
	Cin vs Pl Session B	−1.331	1.234	0.2833	0.8499
	CF vs Pl Session A	0.552	1.44	0.7023	1
	CF vs Pl Session B	−3.072	1.235	0.0145	0.0435
	Cap vs Pl Overall*				0.2025
	Cin vs Pl Overall*				0.4675
	CF vs Pl Overall*				0.0373

Comparison of ANS parameters expressed as differences of AUC over recording sessions.*overall F-test.

The p-values were adjusted for multiple comparison according to Bonferroni.

Abbreviations: Pl, placebo; Cap, capsaicin; Cin, cinnamaldehyde; CF, cooling flavor.

n = 16–18 (see also supplementary Tables S1-S6).

**Table 3:** 

	Comparisons	Difference	SE	Raw p-value	Adjusted p-value
**Systolic**	Cap vs Pl	182.991	120.843	0.1371	0.4113
	Cin vs Pl	−57.401	117.202	0.6267	1
	CF vs Pl	55.96	116.153	0.6323	1
**Diastolic**	Cap vs Pl	−35.859	135.685	0.7928	1
	Cin vs Pl	−198.737	128.191	0.1282	0.3846
	CF vs Pl	−233.805	131.103	0.0814	0.2442
**Mean arterial pressure**	Cap vs Pl	24.677	112.72	0.8277	1
	Cin vs Pl	−148.225	107.865	0.1763	0.5289
	CF vs Pl	−137.87	108.823	0.2119	0.6357

Comparison of blood pressures expressed as difference of AUC over recording sessions.The p-values were adjusted for multiple comparison according to Bonferroni.

Abbreviations: Pl, placebo; Cap, capsaicin; Cin, cinnamaldehyde; CF, cooling flavor.

n = 16–18 (see also supplementary Tables S7-S9).

**Table 4:** 

Zone	Comparisons	Difference	SE	Raw p-value	Adjusted p-value
1	Cap vs Pl	18.584	10.377	0.0809	0.2427
	Cin vs Pl	22.396	10.187	0.0338*	0.1014
	CF vs Pl	5.097	10.196	0.6198	1
2	Cap vs Pl	54.439	35.154	0.1294	0.3882
	Cin vs Pl	42.064	34.458	0.2293	0.6879
	CF vs Pl	24.708	34.551	0.4787	1
3	Cap vs Pl	16.959	13.911	0.2299	0.6897
	Cin vs Pl	30.816	13.906	0.0324*	0.0972
	CF vs Pl	10.554	13.894	0.4519	1
4	Cap vs Pl	14.893	14.793	0.3201	0.9603
	Cin vs Pl	22.118	14.637	0.1386	0.4158
	CF vs Pl	7.862	14.645	0.5943	1
5	Cap vs Pl	8.126	9.672	0.4058	1
	Cin vs Pl	14.065	9.562	0.1491	0.4473
	CF vs Pl	5.246	9.546	0.5857	1
6	Cap vs Pl	13.186	9.899	0.1904	0.5712
	Cin vs Pl	21.144	9.763	0.0363*	0.1089
	CF vs Pl	5.128	9.75	0.6018	1
7	Cap vs Pl	9.255	10.155	0.3676	1
	Cin vs Pl	18.856	10.075	0.0686	0.2058
	CF vs Pl	4.157	10.047	0.6813	1
8	Cap vs Pl	12.469	11.054	0.266	0.798
	Cin vs Pl	18.384	10.881	0.0989	0.2967
	CF vs Pl	2.828	10.879	0.7962	1
9	Cap vs Pl	26.056	13.146	0.0544	0.1632
	Cin vs Pl	18.947	12.902	0.1498	0.4494
	CF vs Pl	15.363	12.901	0.2407	0.7221
10	Cap vs Pl	25.26	13.943	0.0775	0.2325
	Cin vs Pl	34.072	13.567	0.0162*	0.0486**
	CF vs Pl	3.486	13.557	0.7984	1
11	Cap vs Pl	3.299	13.226	0.8043	1
	Cin vs Pl	18.237	13.175	0.174	0.522
	CF vs Pl	−1.749	13.188	0.8952	1
12	Cap vs Pl	22.575	11.895	0.065	0.195
	Cin vs Pl	26.571	11.691	0.0285*	0.0855
	CF vs Pl	9.377	11.693	0.4273	1

Comparisons of face temperature per zone expressed as difference of AUC over recording sessions. Abbreviations: Cap, capsaicin; Cin, cinnamaldehyde; CF, cooling flavor.

The p-values were adjusted for multiple comparisons according to Bonferroni.

*p-value < 0.05; **Adj p-value < 0.05, n = 16–18.

## References

[b1] Mitchell N. S., Catenacci V. A., Wyatt H. R. & Hill J. O. Obesity: overview of an epidemic. Psychiatr. Clin North Am 34, 717–732 (2011). 22098799 10.1016/j.psc.2011.08.005 PMC3228640

[b2] Donahoo W. T., Levine J. A. & Melanson E. L. Variability in energy expenditure and its components. Curr Opin. Clin Nutr Metab Care 7, 599–605 (2004). 15534426 10.1097/00075197-200411000-00003

[b3] Hursel R. & Westerterp-Plantenga M. S. Thermogenic ingredients and body weight regulation. Int. J. Obes. (Lond) 34, 659–669 (2010). 20142827 10.1038/ijo.2009.299

[b4] Ludy M. J., Moore G. E. & Mattes R. D. The effects of capsaicin and capsiate on energy balance: critical review and meta-analyses of studies in humans. Chem. Senses 37, 103–121 (2012). 22038945 10.1093/chemse/bjr100 PMC3257466

[b5] Matsumoto T. *et al.* Effects of capsaicin-containing yellow curry sauce on sympathetic nervous system activity and diet-induced thermogenesis in lean and obese young women. J Nutr Sci. Vitaminol. (Tokyo) 46, 309–315 (2000). 11227803 10.3177/jnsv.46.309

[b6] Yoshioka M. *et al.* Effects of red-pepper diet on the energy metabolism in men. *J Nutr Sci*. Vitaminol. (Tokyo) 41, 647–656 (1995). 10.3177/jnsv.41.647 8926537

[b7] Yoshioka M., St-Pierre S., Suzuki M. & Tremblay A. Effects of red pepper added to high-fat and high-carbohydrate meals on energy metabolism and substrate utilization in Japanese women. Br. J Nutr 80, 503–510 (1998). 10211048 10.1017/s0007114598001597

[b8] Yoshioka M. *et al.* Effects of red pepper on appetite and energy intake. Br. J. Nutr. 82, 115–123 (1999). 10743483

[b9] Yoshioka M., Doucet E., Drapeau V., Dionne I. & Tremblay A. Combined effects of red pepper and caffeine consumption on 24 h energy balance in subjects given free access to foods. Br. J Nutr 85, 203–211 (2001). 11242488 10.1079/bjn2000224

[b10] Lim K. *et al.* Dietary red pepper ingestion increases carbohydrate oxidation at rest and during exercise in runners. Med. Sci. Sports Exerc. 29, 355–361 (1997). 9139174 10.1097/00005768-199703000-00010

[b11] Watanabe T., Kawada T. & Iwai K. Effect of capsaicin pretreatment on capsaicin-induced catecholamine secretion from the adrenal medulla in rats. Proc. Soc. Exp. Biol. Med. 187, 370–374 (1988). 3347612 10.3181/00379727-187-3-rc1

[b12] Caterina M. J. *et al.* The capsaicin receptor: a heat-activated ion channel in the pain pathway. Nature 389, 816–824 (1997). 9349813 10.1038/39807

[b13] Iida T. *et al.* TRPV1 activation and induction of nociceptive response by a non-pungent capsaicin-like compound, capsiate. Neuropharmacology 44, 958–967 (2003). 12726827 10.1016/s0028-3908(03)00100-x

[b14] Zhang L. L. *et al.* Activation of transient receptor potential vanilloid type-1 channel prevents adipogenesis and obesity. Circ. Res 100, 1063–1070 (2007). 17347480 10.1161/01.RES.0000262653.84850.8b

[b15] Lee E. *et al.* Transient receptor potential vanilloid type-1 channel regulates diet-induced obesity, insulin resistance, and leptin resistance. FASEB J 29, 3182–3192 (2015). 25888600 10.1096/fj.14-268300 PMC4511197

[b16] Vetter I. & Lewis R. J. Natural product ligands of TRP channels. Adv. Exp. Med. Biol. 704, 41–85 (2011). 21290289 10.1007/978-94-007-0265-3_3

[b17] Story G. M. *et al.* ANKTM1, a TRP-like channel expressed in nociceptive neurons, is activated by cold temperatures. Cell 112, 819–829 (2003). 12654248 10.1016/s0092-8674(03)00158-2

[b18] Bautista D. M. *et al.* The menthol receptor TRPM8 is the principal detector of environmental cold. Nature 448, 204–208 (2007). 17538622 10.1038/nature05910

[b19] Masamoto Y., Kawabata F. & Fushiki T. Intragastric administration of TRPV1, TRPV3, TRPM8, and TRPA1 agonists modulates autonomic thermoregulation in different manners in mice. Biosci. Biotechnol. Biochem. 73, 1021–1027 (2009). 19420725 10.1271/bbb.80796

[b20] Yoshida T., Yoshioka K., Wakabayashi Y., Nishioka H. & Kondo M. Effects of capsaicin and isothiocyanate on thermogenesis of interscapular brown adipose tissue in rats. J Nutr Sci Vitaminol. (Tokyo.) 34, 587–594 (1988). 3244046 10.3177/jnsv.34.587

[b21] Iwasaki Y., Tanabe M., Kobata K. & Watanabe T. TRPA1 agonists–allyl isothiocyanate and cinnamaldehyde–induce adrenaline secretion. Biosci. Biotechnol. Biochem. 72, 2608–2614 (2008). 18838811 10.1271/bbb.80289

[b22] Batt R. A. & Hambi M. Development of the hypothermia in obese mice (genotype ob/ob). Int J Obes 6, 391–397 (1982). 7129751

[b23] Liu X. *et al.* Brown Adipose Tissue Transplantation Reverses Obesity in Ob/Ob Mice. Endocrinology. 156, 2461–2469 (2015). 25830704 10.1210/en.2014-1598

[b24] Camacho S. *et al.* Anti-obesity and anti-hyperglycemic effects of cinnamaldehyde via altered ghrelin secretion and functional impact on food intake and gastric emptying. Sci Rep 5, 7919 (2015). 25605129 10.1038/srep07919 PMC4300502

[b25] Ma S. *et al.* Activation of the cold-sensing TRPM8 channel triggers UCP1-dependent thermogenesis and prevents obesity. J. Mol. Cell Biol. 4, 88–96 (2012). 22241835 10.1093/jmcb/mjs001

[b26] Ye L. *et al.* Fat cells directly sense temperature to activate thermogenesis. Proc. Natl. Acad. Sci USA 110, 12480–12485 (2013). 23818608 10.1073/pnas.1310261110 PMC3725077

[b27] Bandell M. *et al.* Noxious cold ion channel TRPA1 is activated by pungent compounds and bradykinin. Neuron 41, 849–857 (2004). 15046718 10.1016/s0896-6273(04)00150-3

[b28] Nakayama K., Goto S., Kuraoka K. & Nakamura K. Decrease in nasal temperature of rhesus monkeys (Macaca mulatta) in negative emotional state. Physiol Behav. 84, 783–790 (2005). 15885256 10.1016/j.physbeh.2005.03.009

[b29] Drummond P. D. Mechanisms of physiological gustatory sweating and flushing in the face. J Auton. Nerv. Syst. 52, 117–124 (1995). 7615894 10.1016/0165-1838(94)00151-9

[b30] Ludy M. J. & Mattes R. D. The effects of hedonically acceptable red pepper doses on thermogenesis and appetite. Physiol Behav. 102, 251–258 (2011). 21093467 10.1016/j.physbeh.2010.11.018 PMC3022968

[b31] Alvarez-Collazo J. *et al.* Cinnamaldehyde inhibits L-type calcium channels in mouse ventricular cardiomyocytes and vascular smooth muscle cells. Pflugers Arch. 466, 2089–99 (2014). 24563220 10.1007/s00424-014-1472-8

[b32] Gregersen N. T. *et al.* Acute effects of mustard, horseradish, black pepper and ginger on energy expenditure, appetite, ad libitum energy intake and energy balance in human subjects. Br. J Nutr 109, 556–563 (2013). 23021155 10.1017/S0007114512001201

[b33] Riera C. E., Vogel H., Simon S. A., Damak S. & le Coutre J. Sensory attributes of complex tasting divalent salts are mediated by TRPM5 and TRPV1 channels. J. Neurosci. 29, 2654–2662 (2009). 19244541 10.1523/JNEUROSCI.4694-08.2009 PMC6666243

[b34] Weir J. B. New methods for calculating metabolic rate with special reference to protein metabolism. J. Physiol 109, 1–9 (1949). 15394301 10.1113/jphysiol.1949.sp004363 PMC1392602

[b35] Livesey G. & Elia M. Estimation of energy expenditure, net carbohydrate utilization, and net fat oxidation and synthesis by indirect calorimetry: evaluation of errors with special reference to the detailed composition of fuels. Am J Clin Nutr 47, 608–628 (1988). 3281434 10.1093/ajcn/47.4.608

[b36] Pagani M. *et al.* Power spectral analysis of heart rate and arterial pressure variabilities as a marker of sympatho-vagal interaction in man and conscious dog. Circ. Res. 59, 178–193 (1986). 2874900 10.1161/01.res.59.2.178

[b37] Matsumoto I., Emori Y., Ninomiya Y. & Abe K. A comparative study of three cranial sensory ganglia projecting into the oral cavity: *in situ* hybridization analyses of neurotrophin receptors and thermosensitive cation channels. Brain Res. Mol. Brain Res. 93, 105–112 (2001). 11589988 10.1016/s0169-328x(01)00129-2

