# Supplementary material for: Effects of TRP channel agonist ingestion on metabolism and autonomic nervous system in a randomized clinical trial of healthy subjects
Source: Sci Rep. 2016 Feb 17;6:20795. doi: 10.1038/srep20795 (PMC4756362; doi:10.1038/srep20795)
Supplement: Supplementary Information [file srep20795-s1.pdf]

Effects of TRP channel agonist ingestion on metabolism and autonomic nervous system in a randomized clinical trial of healthy subjects

Stéphanie Michlig [1], Jenny Meylan Merlini[1], Maurice Beaumont[1], Mirko Ledda[1], Aude Tavenard[1], Rajat Mukherjee[1], Susana Camacho[1] and Johannes le Coutre[1,2]

Supplementary information:

Heart rate variability

Tables S1 to S6 represent the distribution of the variables of power spectra analysis of heart rate variability collected in session A and B and averaged over 10 min intervals. The data are expressed for each, SNS index, PNS index and VLF/TP, as the mean (SD).
(n=16-18 for each group; abbreviations: Pl, placebo; CF, cooling flavor; Cap, capsaicin; Cin, cinnamaldehyde).

Table S1.Session A: SNS index expressed as mean (SD)

	time (min)	Pl	CF	Cap	Cin
Before ingestion	0-10	9.7 (7.81)	11.35 (7.96)	7.65 (6.26)	8.87 (5.92)
	10-20	8.91 (5.79)	10.04 (6.61)	9.39 (6.42)	10.15 (6.21)
	20-30	12.25 (9.77)	14.54 (10.25)	10.04 (6.71)	9.85 (6.78)
After ingestion	45-55	10.68 (8.76)	13.97 (10.01)	11.55 (6.73)	9.8 (5.42)
	55-65	10.94 (7.03)	14.53 (11.01)	12.06 (11.79)	11.07 (6.37)
	65-75	13.23 (8.58)	11.9 (7.16)	13.57 (9.44)	10.64 (6.31)
	75-85	11.71 (8.32)	11.72 (6.27)	12.76 (8.92)	10.71 (7.07)
	85-95	13.58 (8.63)	12.82 (6.63)	13.45 (9.52)	9.52 (7)
	95-105	14.68 (13.73)	12.94 (8.05)	11.48 (5.95)	10.78 (5.84)
	105-115	13.51 (9.31)	12.42 (7.29)	14.04 (7.54)	12.16 (10.23)

Table S4. Session B: SNS index expressed as mean (SD)

	time (min)	Pl	CF	Cap	Cin
Before ingestion	0-10	7.72 (4.62)	6.19 (5.21)	6.96 (6.7)	7.05 (4.56)
	10-20	7.54 (4.45)	6.88 (3.81)	6.33 (3.76)	7.51 (4.92)
	20-30	9.46 (5.01)	7.99 (4.09)	8.16 (5.89)	8.77 (6.04)
After ingestion	45-55	7.32 (3.56)	6.82 (4.58)	7.31 (5.01)	7.56 (5.03)
	55-65	8.29 (2.76)	5.77 (2.66)	8.34 (4.39)	8.65 (4.88)
	65-75	9.68 (4.99)	7.74 (4.07)	7.38 (3.36)	9.59 (5.57)
	75-85	11.8 (6.45)	9.61 (6.77)	8.85 (3.5)	11.62 (5.08)
	85-95	13.09 (7.67)	9.79 (6.38)	8.65 (4.96)	10.62 (6.5)
	95-105	12.97 (9.39)	10.06 (7.24)	9.39 (5.6)	9.92 (5.83)
	105-115	13.25 (7.35)	10.42 (6.55)	8.69 (4.53)	10.4 (6.65)

Table S2. Session A: PNS index expressed as mean (SD)

	time (min)	Pl	CF	Cap	Cin
Before ingestion	0-10	0.15 (0.087)	0.148 (0.119)	0.165 (0.070)	0.158 (0.079)
	10-20	0.158 (0.082)	0.154 (0.089)	0.136 (0.050)	0.139 (0.069)
	20-30	0.136 (0.102)	0.111 (0.064)	0.137 (0.063)	0.144 (0.073)
After ingestion	45-55	0.136 (0.063)	0.105 (0.056)	0.116 (0.044)	0.15 (0.088)
	55-65	0.138 (0.092)	0.115 (0.068)	0.141 (0.089)	0.128 (0.070)
	65-75	0.113 (0.081)	0.12 (0.054)	0.113 (0.057)	0.131 (0.060)
	75-85	0.128 (0.094)	0.117 (0.053)	0.123 (0.058)	0.136 (0.076)
	85-95	0.116 (0.070)	0.108 (0.054)	0.117 (0.062)	0.16 (0.098)
	95-105	0.117 (0.089)	0.116 (0.061)	0.117 (0.055)	0.142 (0.100)
	105-115	0.124 (0.093)	0.112 (0.045)	0.113 (0.067)	0.143 (0.089)

Table S5. Session B: PNS index expressed as mean (SD)

	time (min)	Pl	CF	Cap	Cin
Before ingestion	0-10	0.15 (0.087)	0.148 (0.119)	0.165 (0.070)	0.158 (0.079)
	10-20	0.158 (0.082)	0.154 (0.089)	0.136 (0.050)	0.139 (0.069)
	20-30	0.136 (0.102)	0.111 (0.064)	0.137 (0.063)	0.144 (0.073)
After ingestion	45-55	0.136 (0.063)	0.105 (0.056)	0.116 (0.044)	0.15 (0.088)
	55-65	0.138 (0.092)	0.115 (0.068)	0.141 (0.089)	0.128 (0.070)
	65-75	0.113 (0.081)	0.12 (0.054)	0.113 (0.057)	0.131 (0.060)
	75-85	0.128 (0.094)	0.117 (0.053)	0.123 (0.058)	0.136 (0.076)
	85-95	0.116 (0.070)	0.108 (0.054)	0.117 (0.062)	0.16 (0.098)
	95-105	0.117 (0.089)	0.116 (0.061)	0.117 (0.055)	0.142 (0.100)
	105-115	0.124 (0.093)	0.112 (0.045)	0.113 (0.067)	0.143 (0.089)

Table S3. Session A: VLF/TP expressed as mean (SD)

	time (min)	Pl	CF	Cap	Cin
Before ingestion	0-10	0.458 (0.091)	0.456 (0.138)	0.424 (0.135)	0.486 (0.117)
	10-20	0.441 (0.125)	0.49 (0.119)	0.463 (0.126)	0.484 (0.110)
	20-30	0.461 (0.145)	0.518 (0.123)	0.456 (0.134)	0.454 (0.139)
After ingestion	45-55	0.453 (0.136)	0.521 (0.119)	0.52 (0.122)	0.49 (0.132)
	55-65	0.482 (0.135)	0.521 (0.120)	0.47 (0.140)	0.506 (0.134)
	65-75	0.498 (0.148)	0.525 (0.144)	0.513 (0.148)	0.507 (0.134)
	75-85	0.458 (0.158)	0.519 (0.127)	0.499 (0.123)	0.512 (0.142)
	85-95	0.503 (0.121)	0.519 (0.129)	0.515 (0.146)	0.497 (0.133)
	95-105	0.505 (0.188)	0.522 (0.109)	0.498 (0.113)	0.466 (0.142)
	105-115	0.523 (0.164)	0.541 (0.085)	0.511 (0.146)	0.499 (0.140)

Table S6. Session B: VLF/TP expressed as mean (SD)

	time (min)	Pl	CF	Cap	Cin
Before ingestion	0-10	0.486 (0.143)	0.422 (0.162)	0.474 (0.151)	0.509 (0.119)
	10-20	0.466 (0.105)	0.476 (0.130)	0.464 (0.149)	0.496 (0.131)
	20-30	0.502 (0.126)	0.489 (0.116)	0.485 (0.141)	0.509 (0.171)
After ingestion	45-55	0.523 (0.121)	0.485 (0.135)	0.492 (0.133)	0.496 (0.133)
	55-65	0.518 (0.097)	0.441 (0.116)	0.499 (0.098)	0.514 (0.131)
	65-75	0.544 (0.129)	0.502 (0.127)	0.495 (0.096)	0.559 (0.117)
	75-85	0.535 (0.157)	0.499 (0.101)	0.524 (0.068)	0.57 (0.096)
	85-95	0.558 (0.132)	0.533 (0.118)	0.498 (0.090)	0.535 (0.145)
	95-105	0.575 (0.107)	0.53 (0.112)	0.549 (0.083)	0.501 (0.129)
	105-115	0.577 (0.091)	0.516 (0.141)	0.527 (0.112)	0.535 (0.166)

Blood pressure

Tables S7 to S9 represent the distribution of blood pressure (BP) variables: diastolic BP, systolic BP and mean arterial pressure, measured every 15 minutes of session B of the protocol (time points: T0,

T15, T30, T42, T57, T72, T87, T102, T117, T132). The data are expressed as the mean (SD). (n=16-18 for each group; abbreviations: Pl, placebo; CF, cooling flavor; Cap, capsaicin; Cin, cinnamaldehyde).

Table S7. Diastolic BP expressed as mean (SD)

	time (min)	Pl	CF	Cap	Cin
Before ingestion	0	76.94 (6.57)	73.5 (6.73)	77.27 (9.68)	71.94 (6.41)
	15	74.56 (6.53)	71.94 (8.23)	75.33 (9.01)	71.24 (6.04)
	30	71.5 (5.01)	75.11 (8.28)	75.47 (9.28)	71.94 (6.23)
After ingestion	42	78.25 (10.19)	76.78 (8.76)	81.33 (9.61)	74.06 (7.37)
	57	72.75 (7.66)	72.89 (7.72)	76 (7.19)	71.94 (8.32)
	72	72.81 (8.18)	72.83 (9.05)	74.73 (8.53)	73.18 (8.53)
	87	74.81 (7.19)	73.78 (8.54)	75.33 (10.15)	73.18 (7.95)
	102	73.19 (7.88)	72.44 (10.18)	74.27 (9.33)	70.53 (7.17)
	117	75.81 (10.81)	74.61 (9.78)	76.2 (6.04)	71.31 (5.51)
	132	77 (8.89)	75.67 (10.36)	77.2 (9.61)	74.35 (9.16)

Table S8. Systolic BP expressed as mean (SD)

	time (min)	Pl	CF	Cap	Cin
Before ingestion	0	118.25 (5.9)	116.78 (7.5)	119.13 (8.81)	119 (8.92)
	15	115.88 (6.05)	116.67 (6.6)	116.53 (8.01)	116.59 (7.83)
	30	117.44 (6.12)	117.94 (8.16)	117.13 (9.61)	117.24 (9.63)
After ingestion	42	120.69 (7.5)	123.44 (8.31)	125.53 (9.36)	119.29 (7.56)
	57	118.63 (5.44)	120.33 (8.37)	120.93 (7.86)	118.24 (7.77)
	72	119.31 (6.43)	117.94 (6.45)	120.53 (8.69)	117.88 (7.76)
	87	119.19 (7.03)	119.61 (7.08)	118.6 (8.02)	118.35 (6.88)
	102	117.88 (5.5)	120.28 (8.68)	121.13 (9.18)	117.59 (7.89)
	117	118.56 (5.74)	119.28 (8.96)	120.73 (7.12)	117.88 (7.39)
	132	121.19 (7.16)	121.61 (9.89)	122.93 (8)	118.65 (9.01)

Table S9. Mean arterial pressure expressed as mean (SD)

	time (min)	Pl	CF	Cap	Cin
Before ingestion	0	90.75 (5.69)	88 (6.21)	91.27 (8.22)	87.63 (6.48)
	15	88.44 (5.16)	86.83 (6.82)	89.07 (7.84)	86.29 (5.84)
	30	86.88 (4.95)	89.33 (7.77)	89.6 (8.57)	87.06 (6.39)
After ingestion	42	92.28 (7.85)	92.39 (7.55)	96 (8.75)	89.12 (6.65)
	57	88 (5.98)	88.78 (7.03)	91 (5.88)	87.41 (7.39)
	72	88.38 (7.34)	87.89 (7.03)	90.07 (7.98)	88.06 (7.15)
	87	89.63 (5.61)	89.17 (6.55)	89.73 (8.65)	88.29 (7.29)
	102	88.13 (6.58)	88.33 (8.96)	90 (8.73)	86.18 (6.89)
	117	90 (8.12)	89.56 (9.18)	91.13 (5.64)	86.81 (4.72)
	132	91.69 (7.81)	90.94 (9.39)	92.47 (8.59)	89.18 (8.13)
